# Is there a gap between recommended and ‘real world’ practice in the management of depression in young people? A medical file audit of practice

**DOI:** 10.1186/1472-6963-12-178

**Published:** 2012-06-27

**Authors:** Sarah E Hetrick, Andrew Thompson, Kally Yuen, Sue Finch, Alexandra G Parker

**Affiliations:** 1 Orygen Youth Health Research Centre, Centre for Youth Mental Health, University of Melbourne, Melbourne, Australia; 2 The National Youth Mental Health Foundation, Centre of Excellence, Melbourne, Australia; 3 Orygen Youth Health, Melbourne, Australia; 4 Statistical Consulting Centre, University of Melbourne, Melbourne, Australia

## Abstract

**Background:**

Literature has shown that dissemination of guidelines alone is insufficient to ensure that guideline recommendations are incorporated into every day clinical practice.

**Methods:**

We aimed to investigate the gaps between guideline recommendations and clinical practice in the management of young people with depression by undertaking an audit of medical files in a catchment area public mental health service for 15 to 25 year olds in Melbourne, Australia.

**Results:**

The results showed that the assessment and recording of depression severity to ensure appropriate treatment planning was not systematic nor consistent; that the majority of young people (74.5%) were prescribed an antidepressant before an adequate trial of psychotherapy was undertaken and that less than 50% were monitored for depression symptom improvement and antidepressant treatment emergent suicide related behaviours (35% and 30% respectively). Encouragingly 92% of first line prescriptions for those aged 18 years or under who were previously antidepressant-naïve was for fluoxetine as recommended.

**Conclusions:**

This research has highlighted the need for targeted strategies to ensure effective implementation. These strategies might include practice system tools that allow for systematic monitoring of depression symptoms and adverse side effects, particularly suicide related behaviours. Additionally, youth specific psychotherapy that incorporates the most effective components for this age group, delivered in a youth friendly way would likely aid effective implementation of guideline recommendations for engagement in an adequate trial of psychotherapy before medication is initiated.

## Background

A range of guidelines exist for depression in young people, including US guidelines for primary care [1] and the practice parameters of the American Academy of Child and Adolescent Psychiatry (AACAP) [2] UK guidelines [3] and most recently an Australian guideline [4]. Key recommendations are consistent across these, advocating sequencing of interventions from low intensity for mild presentations to more intensive psychotherapy for moderate to severe presentations, with medication (fluoxetine first line) considered if necessary; and in this case close monitoring, particularly for emergent suicidality. However, it is widely recognized that implementation of guideline recommendations into every day clinical practice is far from universal [5] due to barriers at levels: 1. the individual clinician (e.g. knowledge, skills, attitudes); 2. the social context in which the clinician works (e.g. patients, colleagues, authorities); and 3. the organizational context (e.g. resources, organizational climate) [6]. Failure to implement recommendations can result in inappropriate, unnecessary or harmful healthcare provision [7]. For youth depression, as well as endeavoring to ensure recovery, the recommendations are critical in addressing safety concerns about antidepressant medication for this age group.

US research based on data submitted to the Food and Drug Administration (FDA) has shown those up to the age of 25 treated with antidepressant medication are more likely to experience an increase in suicidal ideation and suicide attempts [8-10] resulting in controversy about what constitutes optimal treatment for youth depression [11-14]. We sought to identify the impact of this on clinicians’ implementation of guideline recommendations in a public youth mental health service. In an initial study [15], we identified potential barriers to implementing these recommendations, including clinician beliefs that the recommendations were not relevant to young people presenting to the service, due to the severity and complexity of presentations with clinicians believing medication was warranted earlier than recommended, and that delivery of psychotherapy was difficult. Barriers also existed to undertaking regular monitoring of young people prescribed medication, including a lack of doctors, a perceived lack of expertise of case managers, and lack of time for systematic monitoring resulting in reliance on a passive approach, dependent on client spontaneous report [15]. Our results were consistent with a US study concluding clinicians wanted more emphasis on the therapeutic relationship, and greater flexibility in implementing guideline recommendations [16].

In this second study, we sought to examine actual practice. On the basis of the findings above we hypothesised that ‘real-world’ practice, while broadly in line with guideline recommendations, might require improvements in some areas. At the time of the study, the Australian guidelines did not exist, therefore, we chose the U.K. NICE guidelines [3] as exemplars of the recommendations made in guidelines internationally.

We focused on four key behaviours recommended in the NICE guideline[3]:

1. That the clinician establishes depressive disorder symptom severity

2. That young people are offered medication if the (moderate to severe) depression is unresponsive after receiving four to six sessions of psychological therapy

3. That the first medication offered is fluoxetine

4. That careful monitoring of emergent suicidality and general progress (interpreted in this study as improvement of depression symptoms) take place once fluoxetine is prescribed, operationalized as once a week for four weeks.

The purpose of the research was to investigate these specific behaviours with the aim to ascertain gaps between guideline recommendations and clinical practice in the management of young people with depression in a public mental health setting.

## Methods

### Setting and sample

In Australia, public mental health services provide free mental health care for those with serious mental disorders. Our study took place at Orygen Youth Health (OYH); a public mental health service for young people aged 15-24 living in the northwestern metropolitan area of Melbourne, Australia. OYH receives referrals from primary services (e.g. general practitioners) and secondary services (e.g. pediatricians), although any referrals are considered, including direct referrals from young people, their caregivers, or places such as education settings. The Youth Access Team (YAT) receives these referrals, undertakes assessment and allocates young people to a clinical program. The clinical programs included the Personal Assessment and Crisis Evaluation (PACE) team for those at high risk of developing a psychotic disorder, the Early Psychosis Prevention and Intervention Clinic (EPPIC) for first episode psychosis, the Youth Mood Clinic (YMC) for moderate/severe depression, the Helping Young People Early (HYPE) clinic for emerging borderline personality disorder, the Intensive Mobile Youth Outreach Service (IMYOS) for outreach treatment to enhance engagement, and the service specific Inpatient Unit (IPU).

### Sample

The study aimed to collect data from 150 consecutive clients who met the following inclusion criteria: they entered a clinical program at OYH for the first time between 1 April and 30 September 2007; they had a diagnosis of depressive disorder (DD), or had been prescribed antidepressants or antipsychotics (the latter included to ensure prescription of this class of medication for depressive disorder was captured) at least once in the first six months from the date of first presentation at OYH (the ‘audit period’).

DD was defined as Depressive Disorder NOS, Major Depressive Disorder Single or Recurrent of any level of severity, with or without psychotic features, or where ‘Depressive Disorder’ or ‘MDE’ or ‘Depression’ or ‘Depression Symptom’s’ was recorded in the medical file. This was established after reviewing the entire medical file.

Of the 221 clients entering an OYH clinical program between 1 April and 30 September 2007, 146 met the inclusion criteria. Of these, 121 (83%) had a diagnosis of depression during the audit period. Their mean age was 18.8 years (SD 2.9); 33.1% were male; none identified as indigenous, although four had ‘unknown’ ethnicity. The eligible clients had been with the assessment team (YAT) or inpatient unit on average 32.0 (SD 21.1) days (range 4 to 141) before allocation to a clinical program. The diagnosis or indication of a diagnosis of depression was recorded in 15 different ways at baseline assessment, the most common notation being “MDE or major depressive episode” (62%) with DSM-IV severity specifiers seldom used (<25%). Only 10 of the 121 clients with a depressive diagnosis during the audit period had a diagnosis of depression alone. The remainder had at least one additional diagnosis with these diagnoses classified into 20 categories.

The local ethics review committee approved the study (Melbourne Health Research and Ethics Committee; reference number 2008.18).

### Data collection

A data collection form or ‘audit’ form was designed specifically for the study, which included extensive instructions on coding for each item, and additional information describing codes for medication and diagnosis. Approximately 20% of the files were audited by visiting psychiatric registrars (n = 4), who were given extensive training in use of the audit form by the Principal author (SH) who audited the majority of files. A project specific database was designed and a research assistant entered all of the data from the audit forms.

### Statistical analysis

Descriptive statistics including means, standard deviations and frequency counts were used to describe the timing and type of interventions used, the medications prescribed and the frequency of monitoring of symptoms and adverse outcomes. Where relevant, data analysis was undertaken according to age i.e. those aged 18 years and under and those aged over 18, given the NICE guidelines [3] apply to those 18 years and under. Minitab (version 16) and Microsoft Excel were used to manipulate data files and to obtain the descriptive statistics.

## Results

### Establishing the severity of depression

Only 61 out of 121 (50.4%) patients with a diagnosis of depression had a baseline assessment of the severity of depression recorded using the service’s mandated tools (Health of Nations Outcome Scales; a UK scale measuring the health and social functioning of people with severe mental illness [17]): HoNOS (item 7 on this scale rates “problems with depressed mood” for >18 years) or HoNOSCA (item 9 on this scale rates “problems with emotional or related symptoms” for <18 years). When measurement of depression severity via other means, largely interview based clinical assessment, was taken into account, a total of 104 (86%) clients had severity recorded.

### Timing of prescription of an antidepressant

Thirty-two (26.4%) young people diagnosed with depression during the audit period were already on an antidepressant at the time of entry to OYH. Of those who were antidepressant naïve on entry, 26 (29.2%) did not receive any medication during the audit period; 55 (61.8%) were prescribed one or more antidepressants during the audit period with 20 receiving an antidepressant alone.

**Figure 1:**
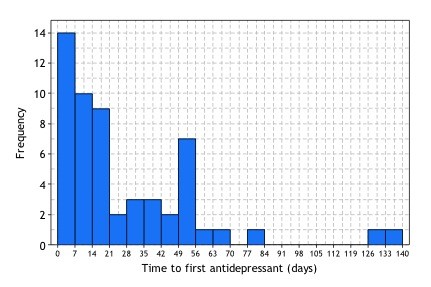
Histogram of the number of days to first anti-depressant prescription (depressed, anti-depressant naïve patients).

The earliest first prescription date was identified for each of the 55 antidepressant naïve clients prescribed an antidepressant during the audit period. The mean number of days to the first prescription of an antidepressant was 27 (SD 29.6); the majority (74.5%; n = 41) receiving this prescription in less than 42 days (see Figure 1) or 6 weeks, about the time it takes to deliver “four to six sessions” of psychological therapy as recommended by the guidelines.

Our earlier work [15] highlighted clinicians’ concerns that severe depression warranted earlier initiation of medication; therefore, we investigated the severity of depression in those who received a prescription for an antidepressant in less than six weeks of entry to OYH (N = 41). Only 33 had a baseline assessment of depression severity recorded (including HoNOS, HoNOSCA or interview based clinical assessment). Of these, 6% had ‘no problem’, 3% had mild depression, 51.5% had moderate depression and 39.4% had severe depression.

### Type of medications prescribed

The first antidepressant prescribed for the 55 antidepressant naïve clients (on entry to OYH) who received a prescription for an antidepressant during the audit period was fluoxetine in 70.9% of cases (92% for those under 18 years of age and 53.3% for those over 18 years of age) (see Table 1).

**Table 1:** Type of first antidepressants for antidepressant naïve patients with a depression diagnosis; overall and by age group

	**All N = 15**	**18 years or younger N = 25**	**Over 18 Years N = 18**
**Medication class**	**Number prescribed this medication**	**Number prescribed this medication**	**Number prescribed this medication**
Citalopram	5 (9.1%)	2 (8.0%)	3 (10.0%)
Escitalopram	3 (5.5%)	0 (0.0%)	3 (10.0%)
Fluoxetine	39 (70.9%)	23 (92.0%)	16 (53.3%)
Paroxetine	2 (3.6%)	0 (0.0%)	2 (6.7%)
Sertraline	2 (3.6%)	0 (0.0%)	2 (6.7%)
Mirtazapine	4 (7.3%)	0 (0.0%)	4 (13.5%)

### Monitoring of emergent suicidality and general progress

As a proxy of the guideline recommendation that young people prescribed medication should be ‘carefully monitored’ (operationalised as ‘weekly…for the first 4 weeks’ (pg.127) [3]), an analysis of how many young people diagnosed with depression and treated with an antidepressant during the audit period (N = 83) had at least one follow-up contact each week for four weeks with any clinician within the first four weeks of prescription of medication was undertaken. Follow-up contact is a contact on any day after the day of prescription of the antidepressant. Contact could be with a doctor, the intake/assessment team (YAT) or an Outpatient Case Manager (OCM), or any combination of these. Contacts with a doctor, for example, were counted as the contacts with a doctor alone or the contacts made in combination with anyone else. Table 2 shows the numbers who had at least one follow-up phone contact each week and at least one follow-up face-to-face contact each week for four weeks.

**Table 2:** Number of patients with one or more successful contacts each week in the four-week follow up after prescription of anti-depressants

	**Any Contact**	**Phone**	**Face-To-Face**
Doctor	1 (1.2%)	0 (0.0%)	1 (1.2%)
OCM	11 (13.3%)	0 (0.0%)	9 (10.8%)
YAT	12 (14.5%)	5 (6.0%)	1 (1.2%)
Any of the above	39 (47.0%)	6 (7.2%)	23 (27.7%)

OCM: Outpatient Case Manager; YAT: Youth Assessment Team.

Twenty-five (30%) of 83 patients with a diagnosis of depression and receiving anti-depressants at some point during the audit period had a suicide ideation/risk assessment at least once a week for four weeks after the first prescription of anti-depressant.

The outcome of the assessment on two questions was considered:

· Any current suicidal ideation/thoughts/intent?

· Any suicide attempt since last contact or current?

Of the 25 patients with four weekly assessments, 10 had a clear answer to the question “Any current suicidal ideation/thoughts/intent?” in every one of the four weeks; for 15 it was unclear from the medical file and therefore recorded as such in the audit form. Only one of the 25 had a clear answer to the question “Any suicide attempt since last contact or current?” in every one of the four weeks.

Twenty-nine (35%) of the 83 were assessed for depression severity at least once per week in the four-week period. Only one of these patients was assessed with a recognized scale.

## Discussion

### Principal findings

As anticipated, based on prior analysis of barriers to implementing youth depression guidelines [15], while practice is broadly in line with guideline recommendations, significant improvements are required in some areas to ensure recommendations are followed appropriately.

First, the current study shows less than 100% concordance with the assessment and recording of depression severity, despite treatment recommendations being largely determined by severity. Depression severity is often simply gauged by clinical judgement using DSM/ICD; however, this was seldom done and even in the context of a mandated tool (though not a specific depression scale), the lack of uniformity in establishing severity striking. In no case was there evidence that a depression scale measure was used.

Second, the majority of young people who were antidepressant naïve on entry to the service did not appear to have an adequate trial of psychological therapy before prescription of an antidepressant. Ours [15] and other research [16] has highlighted clinicians’ concerns about the applicability of evidence for those with severe presentations. The US primary care guidelines (Recommendation 3, pg 1319) [1], practice parameters of the AACAP (Recommendation 9, pg 1511) [2] and new Australian guidelines include a caveat allowing earlier prescription of medication in this case (Recommendation 5: pg 55) [4]. However, the early prescription demonstrated in the current study was not entirely accounted for by the severity of depression.

It could be that medication was prescribed earlier for young people who declined to engage in psychological therapy. Our previous work has highlighted difficulties engaging young people with severe and complex presentations in psychotherapy. Again the US primary care guidelines (Recommendation 3, pg 1319) [1] and practice parameters of the AACAP (Recommendation 9, pg 1511) [2], the Australian guidelines (Recommendation 5, pg 55) [4] as well as the NICE guidelines (Recommendation 7.10.1.3, pg 127) [3] allow provision for medication in this case. This is somewhat paradoxical given the need for close monitoring in the case of medication prescription and the significant gaps between recommendations about monitoring and actual practice highlighted in this research.

Third, fluoxetine was the first medication prescribed for 92% for those 18 years and under, and 53.3% for those over 18 years. This is an encouraging result for those under 18 and suggests that unambiguously stated and potentially less complex recommendations are more likely to be carried out. The result for those over 18 was not unexpected given the audit was undertaken before the Australian guidelines [4], which include ‘good practice points’ recommending extrapolation of evidence for those up to the age of 25, were released. These good practice points are based on the FDA data showing an increased risk of suicide related behaviours after prescription of an antidepressant for those up to the age of 25 [18].

Finally, the Nice guidelines recommend “*careful monitoring of adverse drug reactions, as well as for reviewing mental state and general progress; for example, weekly contact with the child or young person and their parent(s) or carer(s) for the first 4 weeks of treatment*” (Recommendation 7.10.1.3 pg 127) for those initiated on an antidepressant*.* Our results show monitoring was undertaken far less frequently than recommended and was not done in a systematic nor precise way. This is consistent with previous US research [19]. Our previous work regarding clinicians’ perceived lack of expertise and time to undertake systematic monitoring, particularly of medication induced adverse side effects [15]. Together with research that has highlighted that a reliance on spontaneous report of side effects does not sufficiently identify all those at risk of suicide [20], the current results provide further impetus for services to invest in systems that allow regular collection of standardized information about adverse effects, emergent suicidality and resolution of depression symptoms, for example, an easily accessible online tool that incorporates all elements requiring monitoring.

### Strengths and weaknesses of the study

We did not formally assess inter-rater reliability; rather we relied on a high level of detailed instruction in the audit form and intensive training. The Principal author also undertook the majority of the audit.

Our results are based on medical files records rather than observation of what actually took place. This limited the set of behaviours that we could investigate; for example, it was not practical to assess the type of psychotherapy e.g. CBT or IPT being conducted as the assessment of this from case notes was felt to be highly inaccurate.

This is the first study to our knowledge assessing this range of key clinician behaviours in a public youth mental health service relative to guideline recommendations for the treatment of depression in young people. Some research has been undertaken to assess the gaps between guideline recommendations and clinical practice [19,21,22], but each focus on only one or two recommendations. Our results are consistent with research on guideline implementation and the quality of depression care in adults. Adherence to guideline recommendations for adult depression is generally low in primary care e.g. [23-25] even when interventions aiming to improve evidence based care are implemented e.g. [26-28]. Of the few studies that have been undertaken in adult mental health specialist settings, adherence is better. For example in a national survey in the US, while appropriate care (defined as the respondent receiving medication or counseling consistent with guidelines) was only received by 19% of those who visited primary care providers, 90% of those visiting mental health specialists received appropriate care [24]. One study looking at more specific indicators of guideline adherence by psychiatrists in private practice showed targeted training about providing guideline concordant care resulted in patients receiving more medication [29]. Another study in psychiatric outpatient services showed adherence with all of a range of indicators of guideline adherence was seen in up to 55% of cases. Of note was the very low adherence to recommendations regarding routine outcome monitoring in the therapeutic phase [30].

## Conclusion

The publication and dissemination of guidelines is not sufficient to ensure that evidence based recommendations are incorporated into every day clinical practice. This research has highlighted some areas that require specific improvement, including the systematic assessment and recording of depressive disorder symptom severity to ensure appropriate treatment planning. These findings are consistent with other research that has been undertaken and is likely to be applicable to youth public mental health services more broadly. Practice system tools are useful for ensuring the uptake of evidence into practice [31]. For young people with depression, tools that allow for the ongoing monitoring of depression symptoms, adverse side effects - in particular treatment emergent suicide related behaviours - are critical given the increased risk of these outcomes for those on antidepressant medication [10,32]. For young people who decline to engage in psychological therapy but nevertheless require medication, ongoing monitoring is critical. There is room for improvement with regard to ensuring an adequate trial of psychological therapy before medication is initiated, highlighting the need to establish the most effective elements of guideline recommended psychotherapy for young people and develop innovative ways to deliver these in ways that are engaging and acceptable to young people.

Finally, identifying and understanding the evidence-practice gaps, as well as barriers to implementing evidence allows the development of targeted strategies to ensure the uptake of evidence based guideline recommendations into everyday practice.

## Competing interests

The authors declare that they have no competing interests.

## Authors’ contributions

SH conceived of the study, participated in the design of the study, undertook the majority of the file audit and wrote the draft manuscript. AT participated in the design and coordination of the study and helped to draft the manuscript. KY participated in the design of the study, including extensive input into the audit form and into the analysis plan, and commented on the manuscript. SF undertook the final analysis, prepared the figures and tables and commented on the manuscript and AP participated in the design and coordination of the study and helped to draft the manuscript. All authors read and approved the final manuscript.

## Pre-publication history

The pre-publication history for this paper can be accessed here:

http://www.biomedcentral.com/1472-6963/12/178/prepub
